# A multi-task EEG emotion recognition method based on emotion-dimension coupling constraints

**DOI:** 10.1038/s41598-025-34211-z

**Published:** 2026-01-02

**Authors:** Guolin Chen

**Affiliations:** Department of General Education, Gandong University, Fuzhou, Jiangxi 344000 China

**Keywords:** Computational biology and bioinformatics, Neuroscience, Psychology, Psychology

## Abstract

Electroencephalography (EEG)-based emotion recognition seeks to enable multidimensional inference of valence, arousal, and dominance (V–A–D) from non-invasive brain signals. However, most existing methods either process each dimension in isolation or adopt single-task pipelines, which underutilize cross-dimensional information and reduce both generalization and physiological interpretability. To overcome these limitations, we propose a multi-task framework with emotion-dimension coupling constraints (MLT-EDCC) that explicitly encodes inter-dimensional priors during end-to-end training. A shared encoder and three task-specific branches are jointly optimized under three complementary constraints: the V–A circular geometric constraint to enforce circumplex structure, the A–D energy alignment constraint to regulate intensity associations, and the V–D correlation constraint to preserve statistical dependencies. This design shifts learning from independent feature extraction to cross-dimensional structure modeling, thereby promoting coherence across valence, arousal, and dominance and enhancing interpretability. Experiments on two benchmark datasets confirm the effectiveness of MLT-EDCC: on DEAP, accuracies reach 97.68%, 97.74%, and 97.41%; on DREAMER, they achieve 96.16%, 95.78%, and 95.96% for valence, arousal, and dominance, respectively. These results demonstrate that embedding psychological and neurophysiological priors as optimizable constraints offers a principled pathway for robust, generalizable, and interpretable multidimensional EEG emotion recognition.

## Introduction

Emotion is a fundamental component of human psychological activity, shaping patterns of thought, decision-making processes, and behavioral responses, and playing a vital role in daily life and social interaction^1,2^. Psychological studies have shown that positive emotions contribute to mental well-being and are closely associated with emotion regulation capacity, social adaptability, and professional achievement^3^. Consequently, emotion recognition has become an important interdisciplinary topic at the intersection of psychology, computer science, and neuroscience. The concept of “affective computing,” introduced by Professor Rosalind Picard, aims to endow machines with the ability to perceive, understand, and respond to human emotions^4^, thereby providing a foundation for natural human–computer interaction^5^. With the rapid adoption of artificial intelligence in areas such as intelligent healthcare, decision support, and environmental perception, accurate and robust emotion decoding has emerged as a key driver of multidisciplinary integration^6^.

Emotional responses are typically expressed through non-physiological and physiological channels^7^. Non-physiological signals, such as facial expressions and vocal prosody^8^, are easily influenced by voluntary control and may not reflect internal emotional states faithfully. In contrast, physiological signals regulated by the central nervous system-particularly electroencephalogram (EEG), electrocardiogram (ECG), and electrooculogram (EOG)-possess higher reliability for emotion recognition due to their inherent resistance to deliberate manipulation^9^. Among these, EEG signals are widely regarded as an ideal medium for emotion recognition due to their millisecond-level temporal resolution, non-invasive acquisition method, and direct reflection of neural activity^10^. The electrophysiological fluctuations generated by neuronal discharges enable real-time capture of emotional shifts^11^. This “non-forgeable” physiological characteristic also grants EEG unique advantages in fields such as brain-computer interfaces and mental disorder assessment^12^.

Traditional EEG-based emotion recognition methods typically follow a two-stage paradigm of “feature extraction and classifier”^13^. Researchers often employ wavelet transform^14^ or power spectral analysis^15^ to manually construct time–frequency features, which are then classified using algorithms such as K-nearest neighbors or support vector machines^14^. Although these approaches can achieve acceptable results in specific scenarios, they are heavily dependent on expert knowledge, struggle with the non-stationarity of EEG signals and inter-subject variability, and thus exhibit limited generalization. More importantly, most traditional methods independently model each emotional dimension, without explicitly capturing the intrinsic relationships among dimensions, and therefore fail to leverage cross-dimensional complementary information.

In recent years, deep learning has brought significant advances to EEG-based emotion recognition^16^. Convolutional neural networks, in particular, have achieved superior performance on several benchmark datasets by virtue of their automatic feature learning capability. For example, Alhagry et al.^17^ applied an LSTM-based model to the DEAP dataset and reported accuracies of 85.45% and 85.65% on the dimensions of valence and arousal, respectively. Cheng et al.^18^ employed a multi-channel deep forest approach on the DREAMER dataset and achieved 89.03%, 90.41%, and 89.89% on the valence, arousal, and dominance dimensions. Despite these improvements, current deep models still face several common challenges. First, the majority of methods continue to adopt dimension-wise training and evaluation strategies, where the optimization objective is confined to a single dimension and ignores the intrinsic couplings among valence, arousal, and dominance established by psychological and neurophysiological studies. As a result, they fail to adequately capture cross-dimensional consistency and structural dependencies. Second, as network depth and module stacking increase, model complexity grows substantially, leading to higher training costs and computational overhead. Third, the widely used cross-entropy loss function has limited expressiveness in constructing sufficiently discriminative feature spaces and cannot effectively constrain inter-dimensional relationships. Finally, physiological noise contamination and uncertainty inference time continue to hinder model stability and practical applicability.

Motivated by these limitations, existing methods have achieved progress in modeling individual emotional dimensions, yet they remain inadequate in terms of cross-dimensional representation, generalization, and physiological plausibility. To address these issues, we propose a multi-task EEG emotion recognition framework driven by coupling constraints. The framework consists of a shared encoder and task-specific branches, enabling end-to-end optimization for the joint prediction of valence, arousal, and dominance. During training, three complementary coupling priors are introduced to explicitly encode structural relations, intensity alignment, and statistical dependence among dimensions as optimizable constraints, thereby incorporating cross-dimensional structure into the representation learning process. This design not only facilitates information sharing across dimensions but also enhances robustness to physiological noise and inter-subject variability without substantially increasing model complexity. Moreover, it improves prediction consistency and neurophysiological interpretability, providing a theoretically grounded and structurally coherent paradigm for multidimensional EEG-based emotion recognition.

The main contributions of our work are as follows:

- Theory-driven modeling paradigm: This work establishes the coupling constraint theory of affective dimensions by systematically integrating psychological and neurophysiological evidence of structural interrelations among valence, arousal, and dominance.
- Systematic multi-task framework: A hierarchical multi-task architecture is developed, in which a shared encoder captures domain-invariant EEG representations while task-specific branches model dimensional differences, enabling joint prediction of V–A–D and reducing negative transfer.
- Cross-dimensional constraint mechanism: Geometric consistency, intensity alignment, and conditional correlation are introduced into the joint optimization objective, transforming theoretical priors into learnable structural losses that enhance representational consistency, discriminability, and neurophysiological interpretability.
- Comprehensive empirical validation: Extensive experiments on public datasets (DEAP and DREAMER) demonstrate consistent improvements over diverse baselines across all emotional dimensions, with additional gains in robustness and cross-subject generalization, confirming both the proposed paradigm’s effectiveness and generalizability.

## Related work

### Coupling constraint theory of affective dimensions

The field of affective computing widely adopts the three-dimensional model comprising valence, arousal, and dominance as the core framework for emotion modeling^19–21^. This system originates from psychological emotion space theory: Russell’s circular structure model revealed the continuous distribution of emotions within the two-dimensional valence-arousal plane^22^, while Bradley and Lang introduced the dominance dimension in the Self-Assessment Manikin (SAM) to refine the quantitative representation of subjective control^23^. This three-dimensional structure combines psychological explanatory power with computational feasibility, establishing itself as the mainstream theoretical foundation for EEG-based emotion recognition^24^.

It is noteworthy that emotional dimensions are not mutually independent but manifest as a multi-system coupled relationship at the neurophysiological level^25–27^. Valence and arousal together constitute the fundamental dimensions of emotional experience, with their interaction pattern showing that high valence–high arousal corresponds to positive states such as joy, while low valence–high arousal is often associated with negative emotions like anxiety or anger^28^. Neuroimaging studies indicate that positive emotional experiences are closely associated with the dominance of activation in the left prefrontal cortex^29^. The amygdala encodes mainly the intensity of arousal, while the anterior cingulate cortex mediates between these two components^30^. EEG evidence indicates enhanced gamma-band synchronization within fronto-limbic networks during affective processing, with increased frontal connectivity and gamma power under high-arousal positive states^31–33^. This pattern aligns with reports of emotion-related EEG coherence and cross-frequency coupling in frontal circuits.

The dominance dimension captures an individual’s subjective sense of control or power within a given context^23^ and demonstrates dynamic bidirectional interactions with arousal. Low-dominance states, such as fear, are typically accompanied by heightened amygdala activation and increased skin conductance responses^34^, whereas high-dominance states, such as confidence, engage dorsolateral prefrontal regions responsible for cognitive regulation and motivational maintenance^35^. Physiologically, this mechanism is jointly mediated by the autonomic and endocrine systems: the hypothalamic–pituitary–adrenal (HPA) axis regulates arousal intensity through cortisol secretion, while the balance of testosterone and serotonin contributes to the modulation of dominance^36,37^. Notably, when individuals perceive sufficient control, negative high-arousal states may be reinterpreted as challenge-related arousal or positive motivation, underscoring the central role of cognitive reappraisal in emotional regulation^38,39^.

Beyond the arousal–dominance link, valence and dominance also exhibit significant coupling. In most contexts, positive affect is associated with a stronger sense of control, while negative affect corresponds to diminished dominance^23^. Anger constitutes a prominent exception: despite its negative valence, it frequently entails high dominance, reflecting its approach-oriented motivational characteristics^40^. Converging evidence from neuroimaging and EEG studies further supports this relationship. Frontal activation has been shown to correlate not only with positive valence but also with elevated dominance^41^. Moreover, high-dominance states are accompanied by enhanced beta-band synchrony across cortical networks, indicating stronger functional integration during affective processing^42^. Together, these findings suggest that valence and dominance are not independent but are dynamically intertwined through cognitive control and motivational systems.

### Multi-task learning

Multi-task learning (MTL) is a representation paradigm in which multiple related objectives are optimized in parallel within a unified model^43^. Its central premise is to exploit the intrinsic associations among tasks, thereby enhancing the robustness and discriminative power of learned features through parameter sharing and joint constraints^44^. Compared with single-task approaches, MTL alleviates overfitting under limited data conditions by imposing shared inductive biases and improving the efficiency of signal utilization^45^. At the same time, cross-task information transfer and complementarity enable the model to capture both common structures and task-specific variations^46^, leading to gains in performance, interpretability, and adaptability across scenarios. In recent years, this paradigm has been validated in diverse domains such as computer vision, natural language processing, and neural signal decoding, where it demonstrates resilience to noise and domain shifts, more controllable optimization, and the flexibility to incorporate domain priors and structural constraints into learning objectives.

From the perspective of architecture and parameter sharing, MTL methods can be broadly categorized into hard parameter sharing (a shared encoder with task-specific branches)^47^, soft sharing (independent networks with cross-task interaction via regularization, routing, or distillation)^48^, and expert- or attention-based frameworks^49^. Each paradigm reflects different trade-offs among task-relatedness, model complexity, and training stability. Compared with soft sharing or expert-based methods, hard sharing offers a simpler structure with greater parameter efficiency^50^, yielding more stable optimization when applied to medium- or small-scale physiological datasets. Moreover, it naturally accommodates the integration of domain priors as structural constraints, thereby improving both performance and neurophysiological plausibility. In addition, hard sharing reduces the uncertainty introduced by gating and routing hyperparameters, which facilitates reproducibility and systematic ablation studies. Its low inference latency and modest memory footprint also make it well-suited for resource-constrained or real-time deployment scenarios.

## Methodology

### Method overiew

**Fig. 1:**
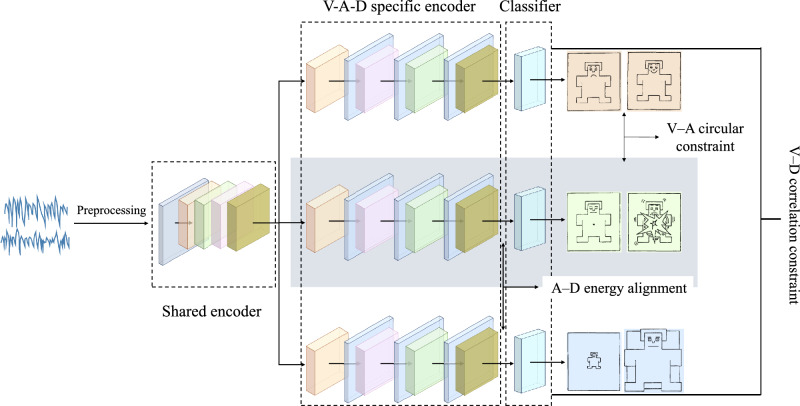
The overall architecture diagram of this paper: the first branch handles valence emotion dimension classification; the second branch corresponds to arousal emotion dimension classification; the third branch is responsible for dominance emotion dimension classification. Three distinct constraint terms are introduced across these branches to model the coupling relationships between dimensions.

This paper proposes a multi-task EEG emotion recognition framework that combines shared representations with emotion-specific dimensions to achieve joint valence, arousal, and dominance prediction. The overall architecture is shown in Fig. 1. It consists of a shared encoder and three task-specific branches. Multi-channel EEG data undergoes preprocessing before inputting into the shared encoder to extract universal representations that simultaneously capture global temporal dependencies and cross-channel context, thereby characterizing stable neurophysiological patterns across subjects. Subsequently, this representation is fed into three task-specific branches, each learning distinct discriminative features for valence, arousal, and dominance. This hierarchical design achieves an effective balance between “shared-specific”: the underlying shared layer enhances data utilization efficiency and reduces redundancy, while the upper-level specific layers suppress negative transfer and enhance dimensional separability. Concurrently, it provides a unified and actionable representation space for cross-dimensional coupling constraints.

To preserve consistency between psychological and neurophysiological predictions in joint forecasting, the training objective incorporates three complementary coupling constraints to structurally regularize outputs and intermediate representations: (1) The V–A circular constraint aligns the geometric distributions of valence and arousal predictions with the quadrant patterns of the circular model, constraining their directional and positional relationships; (2) A–D energy alignment uses feature activation norm as an indicator, requiring synchronized intensity in high-arousal contexts to simulate physiological mechanisms where arousal levels and sense of dominance co-vary; (3) V–D correlation constraint maintains conditional correlation between valence and dominance in the affect prediction space, reflecting the statistical tendency of high pleasantness accompanying high sense of control while allowing context-dependent deviations. These three constraints characterize dimensional coupling through geometric structure, intensity matching, and statistical dependency pathways, respectively. They explicitly inject domain-specific prior knowledge into the optimization process without altering the core architecture.

The design’s uniqueness lies in modular synergy: a shared encoder provides domain-invariant EEG baseline representations, task branches ensure dimension-specificity and discriminative power, while coupling constraints continuously enforce cross-dimensional consistency during training, preventing structural mismatches from purely parallel predictions. Consequently, the model achieves a constrained trade-off between commonality and differentiation, unifying parameter efficiency, optimization stability, and interpretability. Compared to conventional single-task approaches or multi-task schemes relying solely on parameter sharing, this framework translates psychological and neurophysiological evidence into learnable objective terms. While maintaining computational and deployment friendliness, it consistently enhances classification performance, cross-subject generalization, and physiological plausibility. This establishes a theoretically grounded and engineering-feasible modeling pathway for multidimensional EEG emotion recognition.

### Model architecture

The network architecture of this study is built upon the VMamba model. This design preserves VMamba’s efficient temporal modeling capabilities while transforming psychological and neurophysiological priors into optimizable constraints. It balances shared and specialized features, enhancing inter-dimensional consistency, cross-subject generalization, and interpretability. In the feature extraction section, the input multi-channel EEG signal ($x \in \mathbb {R}^{B \times w \times h}$) undergoes standard preprocessing before being fed into the shared encoder. The shared encoder ($\mathscr {E}_{\textrm{sh}}()$) consists of a patch embedding layer and four sequentially stacked VSS (visual state-space) modules, enabling capture of global temporal dependencies and cross-channel contextual information. It outputs a unified general representation denoted as $F_{\textrm{sh}}=\mathscr {E}_{\textrm{sh}}(x)$. Based on this, three independent branches are constructed for valence, arousal, and dominance, respectively. Each branch’s task-specific encoder ($\mathscr {E}_{\textrm{V}}()$, $\mathscr {E}_{\textrm{A}}()$, $\mathscr {E}_{\textrm{D}}()$) adopts a four-level architecture: the first layer is a single VSS block, while the subsequent three layers each consist of a “downsampling + VSS block” unit. This hierarchical design progressively expands the receptive field and enhances abstractive capabilities, ultimately generating more specialized high-level feature representations at the end of each branch, denoted as $F_V= \mathscr {E}_V\!\big (F_{\textrm{sh}}\big )$, $F_A= \mathscr {E}_A\!\big (F_{\textrm{sh}}\big )$, and $F_D =\mathscr {E}_D\!\big (F_{\textrm{sh}}\big )$.

To ensure consistency between classification prediction and cross-dimensional modeling, each branch incorporates a lightweight *FeatureHead* module (comprising normalization, global average pooling, and dimension reduction layers), denoted as $\phi _V()$, $\phi _A()$ and $\phi _D()$, to compress high-dimensional feature maps into low-dimensional vector representations, denoted as $f_V = \phi _V(F_V), f_A = \phi _A(F_a), f_D = \phi _D(F_d)$. This module aims to provide a unified dimensional embedding representation for subsequent cross-dimensional constraint computations. Its structure and function are distinct from the classifier and do not participate in classification scoring. The three branch classifiers ($\mathscr {H}_V$, $\mathscr {H}_A$ and $\mathscr {H}_D$) share identical structures but do not share parameters. Each includes normalization, dimension reshaping, adaptive average pooling, flattening, and fully connected layers, ultimately outputting binary classification probabilities denoted as $P_V = \mathscr {H}_V(F_V), P_A= \mathscr {H}_A(F_A), P_D= \mathscr {H}_D(F_D)$.

At the research objective level, this framework innovates by introducing three types of coupled constraints based on psychological and neurophysiological priors, thereby translating theoretical evidence into optimizable regularization terms. First, the V-A circular geometric constraint acts upon the predicted probability vector $V_p=[P_V,P_A]^{\top }$, ensuring model outputs adhere to the quadrant rules of the affective circular model within a two-dimensional plane, thereby enhancing the psychological plausibility of predictions. The constraint relationship between the valence and arousal dimensions is shown in Eq. (1):

1 $$\begin{aligned} \left\{ \begin{aligned} V_p&= \begin{bmatrix} P_V \\ P_A \end{bmatrix},\\ V_t&= \begin{bmatrix} \hat{P}_V \\ \hat{P}_A \end{bmatrix},\\ \mathscr {L}_{V\!-\!A}&= 1 - \frac{ V_p^\top V_t }{ \Vert V_p\Vert _2 \, \Vert V_t\Vert _2 + \varepsilon } \,. \end{aligned} \right. \end{aligned}$$

Secondly, the A-D energy alignment constraint is based on the norm difference between vector features $(f_A, f_D)$, requiring consistent activation intensity between the two under high arousal states. This simulates the physiological mechanism of coordinated changes in arousal and dominance within the nervous system. The constraint relationship between arousal and dominance dimensions is shown in Eq. (2):

2 $$\begin{aligned} \mathscr {L}_{A\!-\!D} = \big ( \Vert f_A\Vert _2 - \Vert f_D\Vert _2 \big )^2 \,. \end{aligned}$$

Third, the V-D correlation constraint is based on the high-level embedding correlation of $(f_V,f_D)$, ensuring that the pattern where positive emotions typically accompany higher control holds true, while allowing reasonable deviations from this rule for specific emotions like anger. The constraint relationship between the valence and dominance dimensions is shown in Eq. (3):

3 $$\begin{aligned} \mathscr {L}_{V\!-\!D} = 1 - \frac{ f_V^\top f_D }{ \Vert f_V\Vert _2 \, \Vert f_D\Vert _2 + \tau } \,. \end{aligned}$$

Three types of constraints jointly regularize the model from three perspectives-spatial distribution, energy magnitude, and statistical dependence-guiding the network to explicitly learn cross-dimensional structural connections during training.

Algorithm 1 demonstrates the complete process of our proposed research methodology. In general, the framework proposed in this study achieves an organic integration of shared and dedicated components in both structural and task-specific dimensions. The shared encoder ensures stability and generalization of cross-dimensional EEG features, while task-specific branches enhance independent discriminative power across dimensions. The three coupling constraints reinforce cross-dimensional consistency and physiological interpretability of predictions. Through this holistic design, the model achieves improved classification performance and establishes a new paradigm for multidimensional EEG emotion recognition, grounded in robust theoretical foundations and practical value.

**Fig. 2:**
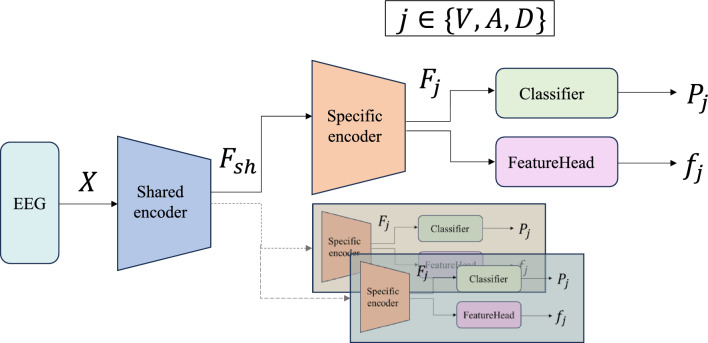
Branch-level architecture for emotion dimension $j$, showing the shared input feature $F_j$ and its parallel processing through the classifier head and the FeatureHead.

**Fig. 3:**
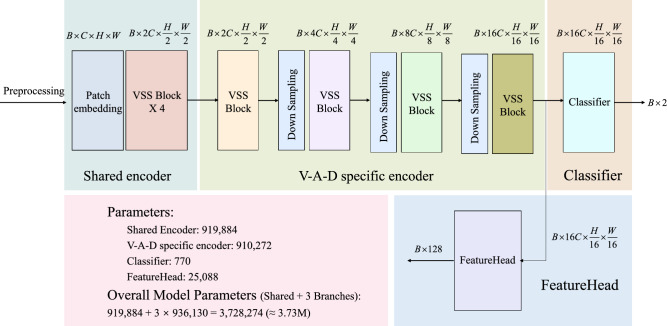
Input and output tensor shapes and learnable parameter counts for each major module in the proposed architecture.

Figure 2, as an auxiliary to Algorithm 1, visually illustrates the detailed structure of a single emotion-dimension branch. It highlights the parallel processing flow of the classifier head and the FeatureHead within each branch, showing how both components share the same input feature map x but serve distinct roles. The FeatureHead plays a critical role in modeling the coupling constraints between emotion dimensions, while the classifier head focuses on generating emotion-specific predictions. This design, as depicted in the figure, reinforces the holistic nature of the model, where classification and constraint satisfaction are integrated to enhance both performance and interpretability. The detailed input/output tensor shapes and parameter counts of each module in the proposed architecture are summarized in Fig. 3.

**Algorithm 1:**
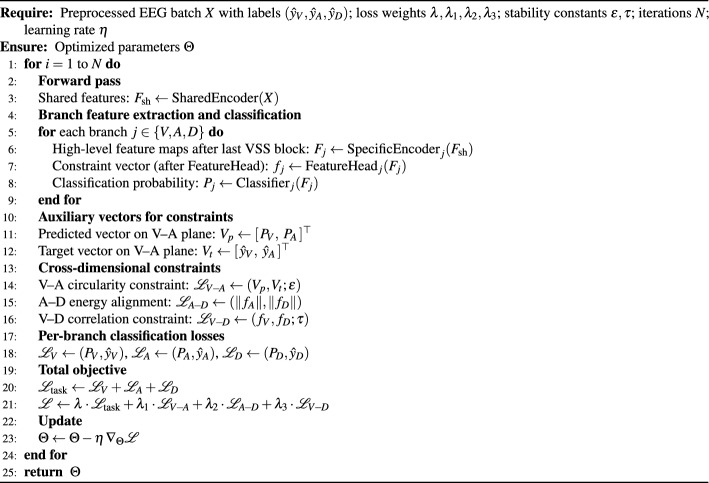
Training pipeline and loss dependencies of the proposed framework.

### Training joint optimization

In multi-task EEG emotion recognition, valence, arousal, and dominance serve as three independent yet interrelated emotional dimensions, each corresponding to a binary classification task. A binary cross-entropy loss function based on Sigmoid activation is introduced at the output end of each task branch to achieve precise discrimination of emotional states across each dimension. This enables supervised learning to assess the degree of matching between model output probabilities and true labels. The cross-entropy loss formula used across the three branches is calculated as follows:

4 $$\begin{aligned} \begin{aligned} \mathscr {L}_V&= - \big [ \hat{y}_V \log P_V + (1-\hat{y}_V)\log (1-P_V) \big ], \\ \mathscr {L}_A&= - \big [ \hat{y}_A \log P_A + (1-\hat{y}_A)\log (1-P_A) \big ], \\ \mathscr {L}_D&= - \big [ \hat{y}_D \log P_D + (1-\hat{y}_D)\log (1-P_D) \big ], \end{aligned} \end{aligned}$$

where $\hat{y}_V, \hat{y}_A, \hat{y}_D \in \{0,1\}$ denote the ground-truth binary labels of valence, arousal, and dominance, respectively; $P_V, P_A, P_D \in (0,1)$ are the predicted probabilities of the three classifiers after the Sigmoid activation.

Building upon this foundation, to simultaneously address discriminative capabilities within dimensions and consistency of inter-dimensional constraints, this paper integrates the three-term cross-entropy loss with the three-category coupling constraint loss into a unified optimization framework. The joint loss function is defined as follows:

5 $$\begin{aligned} \mathscr {L} \;=\; \lambda \big (\mathscr {L}_V + \mathscr {L}_A + \mathscr {L}_D\big ) + \lambda _1 \mathscr {L}_{V\!-\!A} + \lambda _2 \mathscr {L}_{A\!-\!D} + \lambda _3 \mathscr {L}_{V\!-\!D}, \end{aligned}$$

where $\mathscr {L}_V, \mathscr {L}_A, \mathscr {L}_D$ are the binary cross-entropy losses corresponding to the three branches; $\mathscr {L}_{V\!-\!A}, \mathscr {L}_{A\!-\!D}, \mathscr {L}_{V\!-\!D}$ denote the three coupling-constraint losses defined in Eqs. (1–3); $\lambda , \lambda _1, \lambda _2, \lambda _3 \ge 0$ are trade-off hyperparameters that balance classification objectives and inter-dimensional regularization.

## Experiment

### Datasets

The DEAP dataset^51^ contains multimodal recordings acquired while 32 participants viewed 40 music video excerpts. EEG and peripheral physiological signals were collected; this study uses the EEG modality only. After each clip, participants rated valence, arousal, dominance, and liking on a 1–9 scale. EEG was down-sampled to 128 Hz during preprocessing. Following common practice, valence/arousal/dominance (V–A–D) labels were binarized using a threshold of 5 (high vs. low). Each trial thus comprises an EEG array and its label. DREAMER^52^ is a multimodal corpus elicited with audio–visual stimuli and includes EEG and ECG. Recordings were obtained from 23 participants (14 male, 9 female) who watched 18 film clips (each lasting 65 to 393 seconds, with an average length of M = 199 seconds). We analyze the EEG signals sampled at 128 Hz from 14 electrodes. After each clip, subjects provided V–A–D self-assessments on a 1–5 scale; labels were binarized with a threshold of 3.

For both datasets, EEG streams were segmented using a 1 s sliding window (128 samples). In DEAP, each subject contributes 40 trials, yielding 40$\times$60=2400 samples of size 32$\times$128 per subject. In DREAMER, clip durations vary; after segmentation the average yield is 3728 samples of size 14$\times$128 per subject. Table 1 summarizes the array shapes and label formats.

**Table 1:** The information of DEAP and DREAMER datasets.

Dataset	Array name	Array shape	Array contents
DEAP	Data	$40 \times 40 \times 8064$	Video/trial $\times$ channel $\times$ data
	Labels	$40 \times 3$	Video/trial $\times$ label
DREAMER	Data	$18 \times 14 \times 25472\ \text {(M)}$	Video/trial $\times$ channel $\times$ data
	Labels	$18 \times 3$	Video/trial $\times$ label

### Experimental settings

The experimental performance is reported as the average accuracy across 10-fold cross-validation, providing a more reliable evaluation. The model is implemented in PyTorch and trained on an NVIDIA GeForce RTX 4090 GPU for 200 epochs. Training is conducted using the AdamW optimizer with a learning rate of 0.025 to minimize the overall loss function.

As shown in Eq. (5), after extensive experimental validation based on a two-stage tuning strategy (including symmetric-line sweeps, fractional factorial exploration, and Bayesian optimization), we found that the best performance was achieved when the weighting parameters were set to $\lambda = 1.0$, $\lambda _{1} = 0.15$, $\lambda _{2} = 0.1$, and $\lambda _{3} = 0.1$. Under this configuration, the model exhibited the most stable optimization process and achieved the highest recognition accuracy across all emotional dimensions.

### Results and analysis

#### Overall comparison with baseline methods

To comprehensively validate the effectiveness of the proposed method, we conducted a large-scale comparative experiment covering representative models from both traditional and deep learning categories. Traditional baselines included multi-layer perceptron (MLP) and support vector machine (SVM)^53^; deep learning models encompassed convolution recurrent attention model (CRAM)^54^, continuous convolution neural network (Cont-CNN)^53^, dynamical graph convolution neural networks (DGCNN)^55^, and Caps-EEGNet^56^. To ensure a fair comparison, all methods are trained and evaluated using exactly the same input dimensions and preprocessing pipeline. The same trial segmentation strategy, label definition and normalization procedure are applied to the raw EEG signals.

**Fig. 4:**
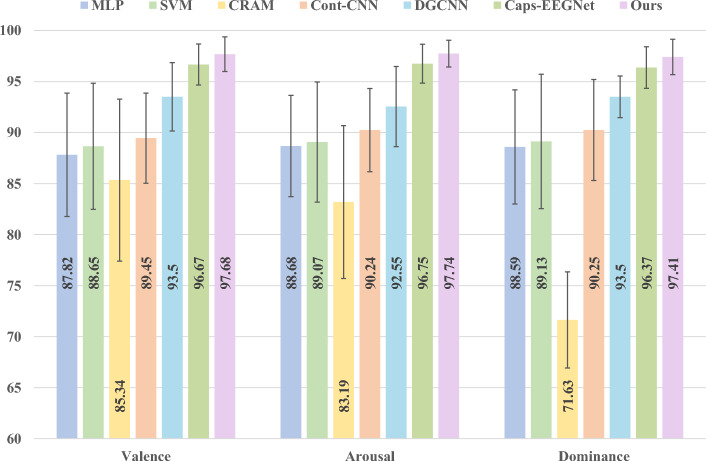
The performance of different contrast methods on the DEAP dataset.

As shown in the experimental results of Table 2, on the DEAP dataset, traditional models (MLP and SVM) exhibit overall limited performance in three-dimensional emotion recognition tasks, particularly struggling to effectively model complex temporal dependencies and spatial features. The optimal traditional baseline SVM achieves accuracy rates of only 88. 65%, 89. 07% and 89. 13% across the dimensions of valence, arousal, and dominance. Under identical data partitioning and training strategies, the proposed method achieves significant improvements in 97.68%, 97.74%, and 97.41%, surpassing the traditional optimal baseline by 9.03, 8.67, and 8.28 percentage points, respectively. Even compared to the state-of-the-art deep learning model Caps-EEGNet (96.67%, 96.75%, 96.37%), our method achieves advantages of 1.01, 0.99, and 1.04 percentage points, accompanied by smaller fluctuation ranges (± 1.70, ± 1.31, ± 1.73). Figure 4 illustrates the performance differences between various methods in the three emotional dimensions. These results demonstrate that our method not only maintains overall accuracy leadership but also exhibits significant advantages in stability and cross-dimensional consistency.

**Table 2:** Comparison of classification accuracy (%) on DEAP dataset across different methods.

Method	Valence	Arousal	Dominance
SVM	88.65 ± 3.42	89.07 ± 3.15	89.13 ± 3.28
MLP	87.62 ± 3.56	88.54 ± 3.44	88.27 ± 3.37
Cont-CNN	89.23 ± 2.85	89.47 ± 2.77	89.65 ± 2.69
CRAM	90.84 ± 2.64	91.32 ± 2.58	90.91 ± 2.61
DGCNN	89.57 ± 2.71	89.83 ± 2.65	90.02 ± 2.63
Caps-EEGNet	96.67 ± 1.92	96.75 ± 1.85	96.37 ± 1.88
Ours	97.68 ± 1.70	97.74 ± 1.31	97.41 ± 1.73

**Fig. 5:**
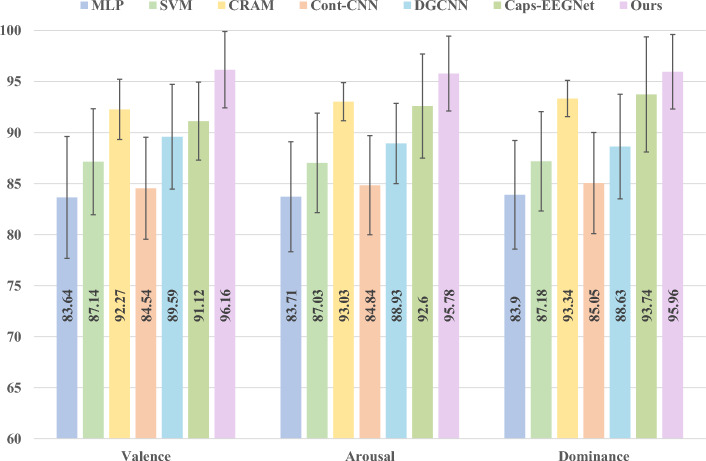
The performance of different contrast methods on the DREAMER dataset.

Experiments on the DREAMER dataset further validate the universality of our approach, with Table 3 detailing the performance metrics. Traditional baselines exhibit accuracy rates primarily concentrated within the 83%–87% range across the three emotion dimensions, with SVM achieving optimal results of 87.14%, 87.03%, and 87.18% respectively, still limited in performance. In contrast, our method achieves 96.16%, 95.78%, and 95.96% on valence, arousal, and dominance, respectively, surpassing the traditional optimal baseline by 9.02, 8.75, and 8.78 percentage points. More critically, even when compared to the state-of-the-art deep learning approach Caps-EEGNet (91.12%, 92.57%, 93.74%), our method maintains leads of 5.04, 3.21, and 2.22 percentage points, while standard deviation remains around ± 3.7. Figure 5 provides a comparison of the results obtained by various methods in the three emotional dimensions. These results fully demonstrate that the proposed model achieves stable and consistent performance gains across different datasets and multidimensional tasks, highlighting its robustness and cross-domain generalization capabilities in EEG emotion recognition.

**Table 3:** Comparison of classification accuracy (%) on DREAMER dataset across different methods.

Method	Valence	Arousal	Dominance
SVM	87.14 ± 4.01	87.03 ± 3.87	87.18 ± 3.92
MLP	85.73 ± 4.25	86.22 ± 4.12	85.96 ± 4.08
Cont-CNN	88.16 ± 3.68	88.45 ± 3.54	88.33 ± 3.49
CRAM	89.77 ± 3.42	90.25 ± 3.37	89.64 ± 3.45
DGCNN	88.92 ± 3.51	89.15 ± 3.46	89.37 ± 3.52
Caps-EEGNet	91.12 ± 3.15	92.57 ± 3.08	93.74 ± 3.02
Ours	96.16 ± 3.74	95.78 ± 3.67	95.96 ± 3.65

#### Ablation study of multi-task and constraints

**Fig. 6:**
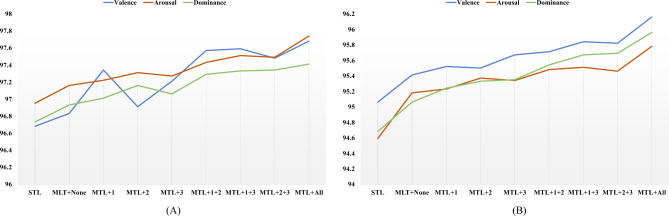
Performance comparison of ablation studies on (**A**) the DEAP dataset and (**B**) the DREAMER dataset. STL denotes the single-task model, while MTL represents the multi-task framework. “+None” indicates the absence of constraints; “+1”, “+2”, and “+3” correspond to the application of the V–A circular geometric constraint, the A–D energy alignment constraint, and the V–D correlation constraint, respectively. Combined notations such as “MTL+1+2” denote the simultaneous use of multiple constraints, and “MTL+All” applies all three constraints.

Additionally, we conducted extensive ablation experiments to validate the progressive impact of the proposed multi-task learning approach and constraint design on EEG emotion recognition performance. Table 4 presents ablation results on the DEAP dataset, where the single-task unconstrained model achieved accuracies of 96.68%/96.95%/96.73% for valence/arousal/dominance recognition, respectively. Introducing multi-task learning without constraints yields modest gains (robust at the 95% confidence level): accuracies increase to 96.83%/97.16%/96.93%, corresponding to improvements of 0.15/0.21/0.20 percentage points. This demonstrates that parameter sharing across dimensions inherently mitigates the limitations of single-dimensional feature representation. Furthermore, the three constraints each emphasize distinct aspects while complementing one another: the V-A circular geometric constraint confines latent representations to a manifold more consistent with the circular distribution of emotions, enhancing the separability of valence and arousal; the A-D energy alignment constraint aligns activation intensity with rhythmic consistency, suppressing classification instability caused by amplitude drift; and the V-D correlation constraint captures the consistency trend between evaluation and dominance, compensating for the blurred boundaries of the dominance dimension. Introducing any single constraint yields stable improvements (for example, valence/arousal reach 97.34%/97.22% under the A–D energy constraint, and dominance reaches 97.16% under the V-D correlation constraint), while any pairwise combination further provides approximately 0.3–0.6 percentage points of additional improvement, demonstrating the complementary regularization effects of structural priors. Applying all three constraints simultaneously yields optimal results of 97.68%/97.74%/97.41%, representing improvements of 1.00/0.79/0.68 percentage points compared with the single-task unconstrained model and 0.85/0.58/0.48 percentage points compared with the multi-task unconstrained model. The performance trends under different constraint strategies are shown in Fig. 6A. The overall results align with the tabular analysis, further illustrating the sustained improvements achieved through the synergistic collaboration of multi-task and multi-constraint approaches.

**Table 4:** Ablation study of different constraints on DEAP dataset (%).

	Methods			Valence	Arousal	Dominance
	V–A circular geometric	A–D energy alignment	V–D correlation			
Single-task	No constraints			96.68	96.95	96.73
Multi-task	$\times$	$\times$	$\times$	96.83	97.16	96.93
	$\checkmark$	$\times$	$\times$	97.34	97.22	97.01
	$\times$	$\checkmark$	$\times$	96.91	97.31	97.16
	$\times$	$\times$	$\checkmark$	97.21	97.27	97.06
	$\checkmark$	$\checkmark$	$\times$	97.57	97.43	97.29
	$\checkmark$	$\times$	$\checkmark$	97.59	97.51	97.33
	$\times$	$\checkmark$	$\checkmark$	97.48	97.49	97.34
	$\checkmark$	$\checkmark$	$\checkmark$	97.68	97.74	97.41

The findings on DREAMER corroborate those on DEAP, highlighting the cross-dataset transferability of the proposed constraints. As summarized in Table 5, the single-task model without constraints attains 95.06%/94.59%/94.68% (valence/arousal/dominance). Switching to multi-task learning without constraints improved performance to 95.41%/95.18%/95.06% (0.35/0.59/0.38), further validating the baseline gains from multi-dimensional collaborative learning. Constraint-specific observations reveal: V–A geometric constraints and A–D energy constraints respectively strengthened the two-dimensional manifold of emotions and amplitude consistency, yielding more direct improvements for valence/arousal. V–D correlation constraints effectively mitigated the loose classification boundaries for dominance (e.g., dominance reached 95.35% after introducing V–D). Pairwise combinations exhibited an approximately additive improvement trend (reaching up to 95.84%/95.51%/95.67%), outperforming single constraints in three dimensions, indicating no significant trade-off conflicts among constraints. The global optimum is achieved with all three constraints: 96.16%/95.78%/95.96%, representing improvements of 1.10/1.19/1.28 over single-task unconstrained models and 0.75/0.60/0.90 over multi-task unconstrained models. The ablation experiment results on the DREAMER dataset are shown in Fig. 6B. It is clearly visible that as constraints are progressively introduced, the accuracy of all three emotion dimensions exhibits a steady upward trend. Combining results from both datasets reveals that these structural constraints, aligned with affect theory and signal characteristics, serve as effective regularization to mitigate overfitting risks. Simultaneously, by guiding latent representations to follow more reasonable geometric and statistical relationships, they consistently deliver stable, reproducible performance improvements. No significant “sacrifice-compensation” effects were observed across different dimensions and data distributions, highlighting the robustness and universality of the proposed multi-task-multi-constraint framework.

**Table 5:** Ablation study of different constraints on DREAMER dataset (%).

	Methods			Valence	Arousal	Dominance
	V–A circular geometric	A–D energy alignment	V–D correlation			
Single-task	No constraints			95.06	94.59	94.68
Multi-task	$\times$	$\times$	$\times$	95.41	95.18	95.06
	$\checkmark$	$\times$	$\times$	95.52	95.23	95.24
	$\times$	$\checkmark$	$\times$	95.50	95.37	95.33
	$\times$	$\times$	$\checkmark$	95.67	95.34	95.35
	$\checkmark$	$\checkmark$	$\times$	95.71	95.48	95.54
	$\checkmark$	$\times$	$\checkmark$	95.84	95.51	95.67
	$\times$	$\checkmark$	$\checkmark$	95.82	95.46	95.69
	$\checkmark$	$\checkmark$	$\checkmark$	96.16	95.78	95.96

#### Hyperparameter sensitivity analysis of the classification loss weight

In our multi-task learning framework, the total loss consists of the cross-entropy loss for emotion classification (weighted by $\lambda$) and three auxiliary constraint losses (weighted by $\lambda _1, \lambda _2, \lambda _3$, respectively). To ensure optimization stability and prioritize the primary classification objective, we fix the auxiliary constraint weights to $\lambda _1 = 0.15$, $\lambda _2 = 0.1$, and $\lambda _3 = 0.1$, based on empirical calibration of loss magnitudes and convergence behavior. This configuration allows the constraints to provide moderate structural guidance without overwhelming the main task gradient. Consequently, the weight $\lambda$ of the classification loss becomes the key tunable hyperparameter governing the model’s learning focus.

To systematically evaluate its impact, we conduct a sensitivity analysis over a broad range $\lambda \in [0.01, 10]$ on both the DEAP and DREAMER datasets. As shown in Table 6 and Table 7, model performance exhibits a pronounced nonlinear response to $\lambda$. When $\lambda = 0.01$, the contribution of the classification loss is severely suppressed, rendering the model unable to learn effective emotion-discriminative features from label supervision, which leads to significant performance degradation (Valence accuracy drops to 75.34% on DEAP and 71.25% on DREAMER). This clearly demonstrates the irreplaceable role of supervised signals in affective recognition. As $\lambda$ increases beyond 0.6, the classification objective dominates the optimization, resulting in rapid performance improvement and entry into a stable plateau. Within the interval $\lambda \in [0.9, 1.4]$, the model achieves consistently high and stable performance. Specifically, the DEAP dataset peaks at $\lambda = 1.0$ (Valence: 97.68%, Arousal: 97.74%, Dominance: 97.41%), while DREAMER shows a marginal gain at $\lambda = 1.1$ (96.17%, 95.78%, 95.99%). However, the maximum difference compared to $\lambda = 1.0$ (96.16%, 95.78%, 95.96%) is merely 0.03 percentage points-statistically and practically negligible. Considering the primacy of the classification task, cross-dataset consistency, and the desire to avoid configuration fragmentation, we adopt $\lambda = 1.0$ as the unified setting for all experiments.

This sensitivity analysis not only highlights the decisive influence of the main loss weight on model behavior but also substantiates the rationality of our chosen hyperparameter in balancing performance, robustness, and generalization.

**Table 6:** Performance (%) on the DEAP dataset under varying values of$\lambda$.

$\lambda$	Valence	Arousal	Dominance
0.01	75.34	74.67	76.26
0.1	87.62	87.28	86.98
0.6	94.54	94.06	94.17
0.9	97.41	97.36	97.23
1.0	97.68	97.74	97.41
1.1	97.62	97.70	97.38
1.4	97.35	97.28	97.21
2.0	97.04	97.26	97.14
5.0	96.92	97.22	97.05
10.0	96.87	97.19	96.99

**Table 7:** Performance (%) on the DREAMER dataset under varying values of$\lambda$.

$\lambda$	Valence	Arousal	Dominance
0.01	71.25	70.38	71.07
0.1	82.91	82.09	82.60
0.6	92.87	92.74	93.01
0.9	95.94	95.61	95.83
1.0	96.16	95.78	95.96
1.1	96.17	95.78	95.99
1.4	96.05	95.63	95.74
2.0	95.83	95.50	95.68
5.0	95.57	95.48	95.59
10.0	95.49	95.23	95.11

#### Computational efficiency analysis

Table 8 shows the time comparison under different training strategies. It can be observed that on both the DEAP and DREAMER datasets, the proposed multi-task framework demonstrates significant improvements in both training and inference time compared to traditional single-task models. Specifically, on the DEAP dataset, the three single-task models required 23 min 9 s for training and 0.96 s for testing, whereas the multi-task framework achieved training in just 10 min 12 s and testing in 0.40 s, representing approximately 56% reduction in training time and 58% reduction in inference time. On the DREAMER dataset, the three single-task models required 19 min 51 s for training and 0.66 s for testing, whereas the multi-task framework achieved training in 8 min 52 s and testing in 0.26 s, representing reductions of approximately 55% and 61%, respectively.

**Table 8:** Time consumption comparison of single-task and multi-task models on DEAP and DREAMER datasets.

Dataset	Dimension	DEAP		DREAMER	
		Training	Testing	Training	Testing
Single-task	V/A/D	7 min 43 s	0.32 s	6 min 37 s	0.22 s
	V&A &D	23 min 9 s	0.96 s	19 min 51 s	0.66 s
Multi-task (Ours)	V&A &D	10 min 12 s	0.4 s	8 min 52 s	0.26 s

The “Dimension” column represents the number of emotion dimensions processed in the emotion recognition task. V/A/D denotes the time required for recognizing a single emotion dimension, while V&A &D indicates the total time required for recognizing all three emotion dimensions.

In summary, the multi-task learning framework proposed in this paper demonstrates significant improvements in both accuracy and efficiency compared to existing methods. By introducing constraints such as emotional dimension coupling and focus quality prediction tasks, our approach not only effectively enhances the model’s performance in multi-dimensional emotion recognition but also strengthens its robustness and generalization capabilities. Although the accuracy gains from each constraint are relatively modest, they are crucial for capturing subtle shifts in sentiment states and enhancing task interpretability, thereby boosting the model’s reliability in practical applications. Simultaneously, the multi-task framework reduces training and inference times by approximately 55% to 60% compared to single-task models, significantly improving computational efficiency and real-time processing capabilities. Overall, the proposed multi-task learning approach demonstrates substantial comprehensive advantages in precision, efficiency, and interpretability, offering an effective solution with practical application value.

#### Additional cross-subject evaluation via LOSOCV

To further examine the robustness of the proposed framework under cross-subject distribution shifts, we additionally conducted a Leave-One-Subject-Out Cross-Validation (LOSOCV) experiment on both the DEAP and DREAMER datasets. In this protocol, the data of each subject were in turn held out as the test set, while the remaining subjects were used for training, which imposes a much stricter requirement on the model’s generalization ability than the within-subject 10-fold CV adopted in the main experiments. The detailed results are summarized in Table 9.

**Table 9:** Comparison of classification accuracy (%) under LOSOCV and 10-fold CV on DEAP and DREAMER datasets.

Dataset	Training settings	Constraints	Valence	Arousal	Dominance
DEAP	LOSOCV	$\times$	67.62	66.59	67.33
	LOSOCV	$\checkmark$	69.18	70.86	70.42
	10-fold CV	$\times$	96.83	97.16	96.93
	10-fold CV	$\checkmark$	97.68	97.74	97.41
DREAMER	LOSOCV	$\times$	64.89	64.10	64.04
	LOSOCV	$\checkmark$	67.53	67.24	66.88
	10-fold CV	$\times$	95.41	95.18	95.06
	10-fold CV	$\checkmark$	96.16	95.78	95.96

On the DEAP dataset, the proposed model without constraints achieves LOSOCV accuracies of 67.62%, 66.59%, and 67.33% for valence, arousal, and dominance, respectively. When the full set of structural constraints is activated, the performance is consistently improved to 69.18%, 70.86%, and 70.42% on the three dimensions, corresponding to gains of 1.56, 4.27, and 3.09 percentage points. Although these accuracies are notably lower than those obtained under the within-subject 10-fold CV setting (97.68%, 97.74%, and 97.41%), the gap of roughly 27–29 percentage points is expected, since LOSOCV explicitly forces the model to generalize to unseen subjects without any subject-specific adaptation. More importantly, the clear improvement brought by the constraints under such a challenging protocol indicates that the proposed priors indeed help the model capture more transferable and subject-invariant emotional representations.

A similar pattern is observed on the DREAMER dataset. Under LOSOCV, the accuracies of the unconstrained model are 64.89%, 64.10%, and 64.04% on valence, arousal, and dominance, respectively, while introducing all three constraints increases the performance to 67.53%, 67.24%, and 66.88%. This yields absolute gains of 2.64, 3.14, and 2.84 percentage points, again demonstrating consistent benefits across all emotional dimensions. Compared with the corresponding 10-fold CV results (96.16%, 95.78%, and 95.96%), the LOSOCV accuracies reflect the intrinsic difficulty of cross-subject EEG emotion recognition on DREAMER, yet the proposed multi-task multi-constraint framework still maintains stable behaviour and non-trivial accuracy levels.

Overall, the LOSOCV experiments on DEAP and DREAMER confirm that: (1) the high accuracies reported under the 10-fold CV setting mainly reflect strong within-subject modelling capability, while cross-subject emotion recognition remains substantially more challenging; and (2) the proposed structural constraints consistently improve performance even in this strict evaluation scenario, suggesting that they act as effective regularizers to enhance cross-subject generalization rather than merely fitting subject-specific patterns.

## Discussion

### Comparative analysis with state-of-the-art methods

**Table 10:** Representative advances in EEG emotion recognition across benchmark datasets.

Author	Model	Features	Dataset	Average accuracy(%)		
				Valence	Arousal	Dominance
Li et al.^57^, 2021	3DFR-DFCN	3-D EEG arrays	DEAP	94.59	95.32	94.78
			DREAMER	93.15	91.30	92.04
Huang et al.^58^, 2021	BiDCNN	EEG Feature Matrix	DEAP	94.38	94.72	-
Sartipi et al.^59^, 2021	GFT-STANN	EEG-based GFT coefficients	DEAP	94.80	96.10	-
Li et al.^60^, 2022	SFCSAN	Frequency feature	DEAP	95.15	95.76	95.64
			DREAMER	93.77	95.80	96.26
Liu et al.^61^, 2023	GLFANet	EEG undirected graph	DEAP	94.53	94.91	95.35
			DREAMER	94.57	94.82	94.51
Sartipi et al.^62^, 2023	STANN	Spatial–spectral characteristics	DEAP	95.6	97.0	-
Zheng et al.^63^, 2024	STS-Transformer	Raw EEG signals	DEAP	89.86	86.83	-
			DREAMER	85.09	82.32	-
Zhou et al.^64^, 2025	MS-iMamba	Raw EEG signals	DEAP	94.69	95.03	-
			DREAMER	94.54	95.34	-
Tajmirriahi et al.^65^, 2025	OMLFA	Statistical and spatial features	DEAP	93.66	93.96	94.27
			DREAMER	93.96	95.32	95.52
Wang et al.^66^, 2025	Att-1DCNN-GRU	Raw EEG signals	DREAMER	95.95	94.93	94.91
Ours	MTL-EDCC	Raw EEG signals	DEAP	97.68	97.74	97.41
			DREAMER	96.16	95.78	95.96

We summarize representative methods in EEG-based emotion recognition research over the past four years, as shown in Table 10. Overall, research progress has primarily focused on two aspects: feature construction and model architecture. On one hand, some studies attempt to enhance discriminative capabilities by constructing high-dimensional feature matrices, graph structures, or frequency-domain features, such as BiDCNN^58^, GFT-STANN^59^, and GLFANet^61^, which generally achieve 94%–96% accuracy on the DEAP dataset, demonstrating the advantage of structured features in enhancing model stability. On the other hand, with the advancement of deep learning, increasing research has shifted toward end-to-end modeling using raw EEG signals, exemplified by STS-Transformer^63^ and MS-iMamba^64^. Although their performance on the DREAMER dataset is less stable than traditional feature-driven methods, they demonstrate potential in modeling temporal dependencies and capturing cross-channel relationships. Notably, recent research trends have evolved from single network architectures toward hybrid frameworks integrating attention mechanisms, graph modeling, and multi-scale representations, exemplified by STANN^62^ and OMLFA^65^, which demonstrate strong generalization capabilities across diverse datasets. In contrast, the proposed multi-task learning with emotion-dimension coupling constraints framework (MTL-EDCC) achieves over 95% accuracy on both the DEAP and DREAMER datasets, demonstrating comprehensive superiority across valence, arousal, and dominance dimensions. This indicates that effectively incorporating affective-dimensional correlation constraints while preserving end-to-end feature learning advantages significantly enhances model performance and robustness. This result not only demonstrates the competitiveness of the proposed method within the existing research landscape but also provides a more universal solution for cross-dataset sentiment recognition. It is worth noting that two recent studies, Lin et al.^67^ and the work of zhong et al.^68^ have made notable progress in cross-subject generalization through deep feature aggregation with hemisphere asymmetry and multi-source domain adaptation, as well as adaptive feature alignment across datasets, respectively. While these approaches differ from our emphasis on emotion-dimension coupling constraints, they highlight the growing importance of domain-invariant and subject-independent modeling in practical affective computing. Improving model generalization under limited labeled data or unseen subjects remains a key challenge, and we envision that integrating domain adaptation techniques with dimensional constraint modeling, as suggested by these works, could be a promising direction for our future research.

### Neurophysiological interpretability and visualization

**Fig. 7:**
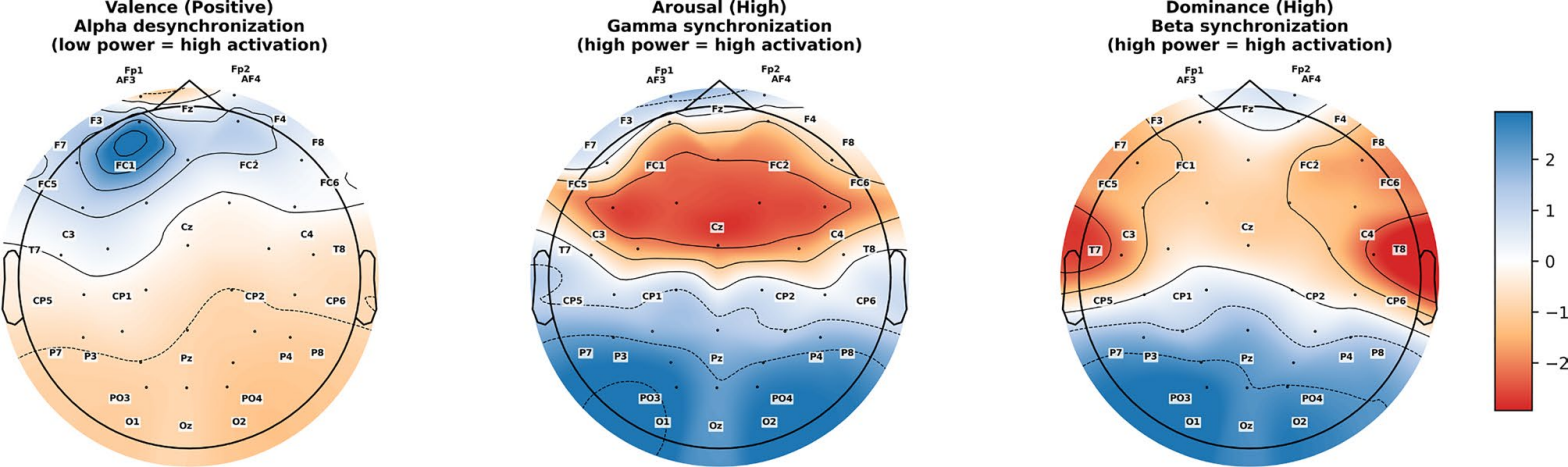
Learned spectral feature topomaps for DEAP dataset. (Left) Positive Valence: Shows distinct Alpha-band desynchronization (blue indicates high activation) in the left frontal region. (Middle) High Arousal: Shows Gamma-band synchronization (red indicates high power) in central–parietal areas. (Right) High Dominance: Shows Beta-band synchronization in right posterior–temporal regions.

**Fig. 8:**
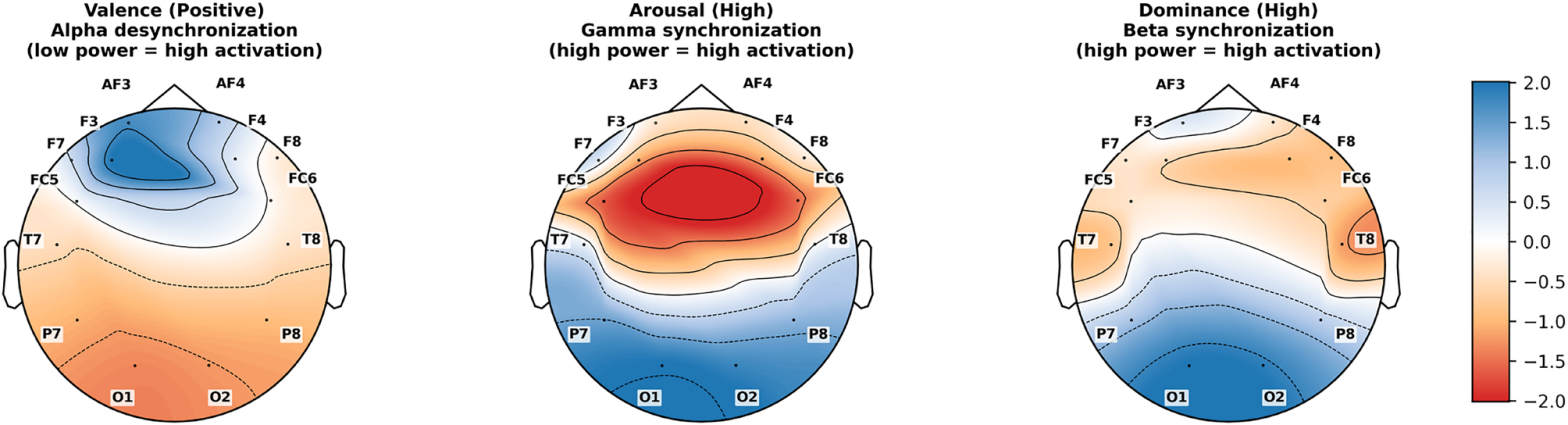
Learned spectral feature topomaps for DREAMER dataset. (Left) Left-frontal Alpha desynchronization for Valence; (Middle) Central Gamma synchronization for Arousal; (Right) Temporal–occipital Beta synchronization for Dominance, demonstrating the robustness of the learned representations.

To further investigate whether the performance gains of the MTL-EDCC framework stem from learning biologically meaningful features, we visualized the spatial and spectral patterns most salient for predicting high valence, arousal, and dominance states. Figure 7 illustrates the learned topomaps from the DEAP dataset (utilizing the 32-channel 10–20 system), while Fig.8 presents the corresponding results from the DREAMER dataset (utilizing the 14-channel system). Although these datasets differ in sensor density and stimulus modality, the observed neural patterns exhibit meaningful consistency with the neurophysiological priors encoded by our coupling constraints.

Regarding the valence dimension, the visualizations in both Fig. 7 (left column) and Fig. 8 (left column) highlight areas of Alpha-band desynchronization predominantly over the left frontal region (e.g., electrodes F3 and FC5 in DEAP; AF3 in DREAMER). This pattern corresponds with the V–A circular geometric constraint and is broadly consistent with the well-established theory of frontal alpha asymmetry, which associates left-lateralized frontal activation with approach-related positive affect. This suggests that the geometric constraint encourages the shared encoder to respect the manifold structure of emotion, effectively leveraging lateralized cortical activation as a discriminative feature.

For high arousal states, the model emphasizes Gamma-band synchronization, particularly centered around the central–parietal cortex (e.g., Cz and CPz), as shown in the middle columns of Figs. 7 and 8. This finding aligns with the objective of the A–D energy alignment constraint, which models the intensity relationship between arousal and dominance. Neurophysiologically, gamma oscillations are often linked to heightened attention and sensory integration during intense emotional processing. The visualization suggests that the energy constraint helps stabilize the model’s focus on these high-frequency dynamics to characterize the intensity of arousal.

In terms of dominance, the model assigns higher importance to Beta-band activity in the right posterior temporal and occipital regions (e.g., T8, P8, and O2), visible in the right columns of the figures. While the neural correlates of dominance are less canonical than those of valence, this pattern tentatively resonates with studies suggesting right-hemisphere involvement in context evaluation and cortical synchronization during affective processing. It implies that the V–D correlation constraint may facilitate the extraction of features related to cognitive control (“sense of agency”), distinguishing them from the broad excitation of arousal.

It is important to note that these interpretations are based on post-hoc analyses of learned features, and individual variability naturally exists across subjects. Nevertheless, the correspondence between the model’s focus regions and established neurophysiological principles provides supportive evidence for the interpretability of the proposed method. It indicates that embedding dimensional coupling priors as optimizable constraints can effectively steer deep networks toward biologically grounded representations, rather than merely fitting spurious statistical correlations.

## Conclusion

This paper addresses the inadequacy of multidimensional correlation modeling in EEG-based emotion recognition by proposing a multi-task learning collaborative framework with emotional dimension coupling constraints (MTL-EDCC). It explicitly introduces structural constraints across the three dimensions of valence, arousal, and dominance on a shared encoder, guiding latent representations with emotion theory and signal characteristics as prior knowledge. Systematic experiments demonstrate that MTL-EDCC achieves leading accuracy across all three dimensions on both the DEAP and DREAMER datasets, accompanied by lower variance and stronger cross-dataset consistency. Ablation results further confirm that multi-task collaboration provides robust baseline gains, while geometric, energy, and correlation constraints complement each other to deliver reproducible marginal improvements within the high-performance range. In summary, the proposed method demonstrates advantages in accuracy, stability, and universality, providing an effective paradigm for structured modeling of multidimensional affective signals. Future work may explore cross-modal information fusion and adaptive individualized modeling for larger-scale and multi-center datasets, enhance subject-independent generalization capabilities, and advance interpretability and clinical/real-world applications.

## Data Availability

The DEAP dataset and the DREAMER dataset are both publicly available. They can be accessed at http://www.eecs.qmul.ac.uk/mmv/datasets/deap/ and https://zenodo.org/records/546113, respectively.
